# Supplementary material for: Inoculation with Plant Growth-Promoting Bacteria and Nitrogen Doses Improves Wheat Productivity and Nitrogen Use Efficiency
Source: Microorganisms. 2023 Apr 17;11(4):1046. doi: 10.3390/microorganisms11041046 (PMC10142644; doi:10.3390/microorganisms11041046)
Supplement: Supplementary file 1 [file microorganisms-11-01046-s001.zip › microorganisms-2286172-supplementary.pdf]

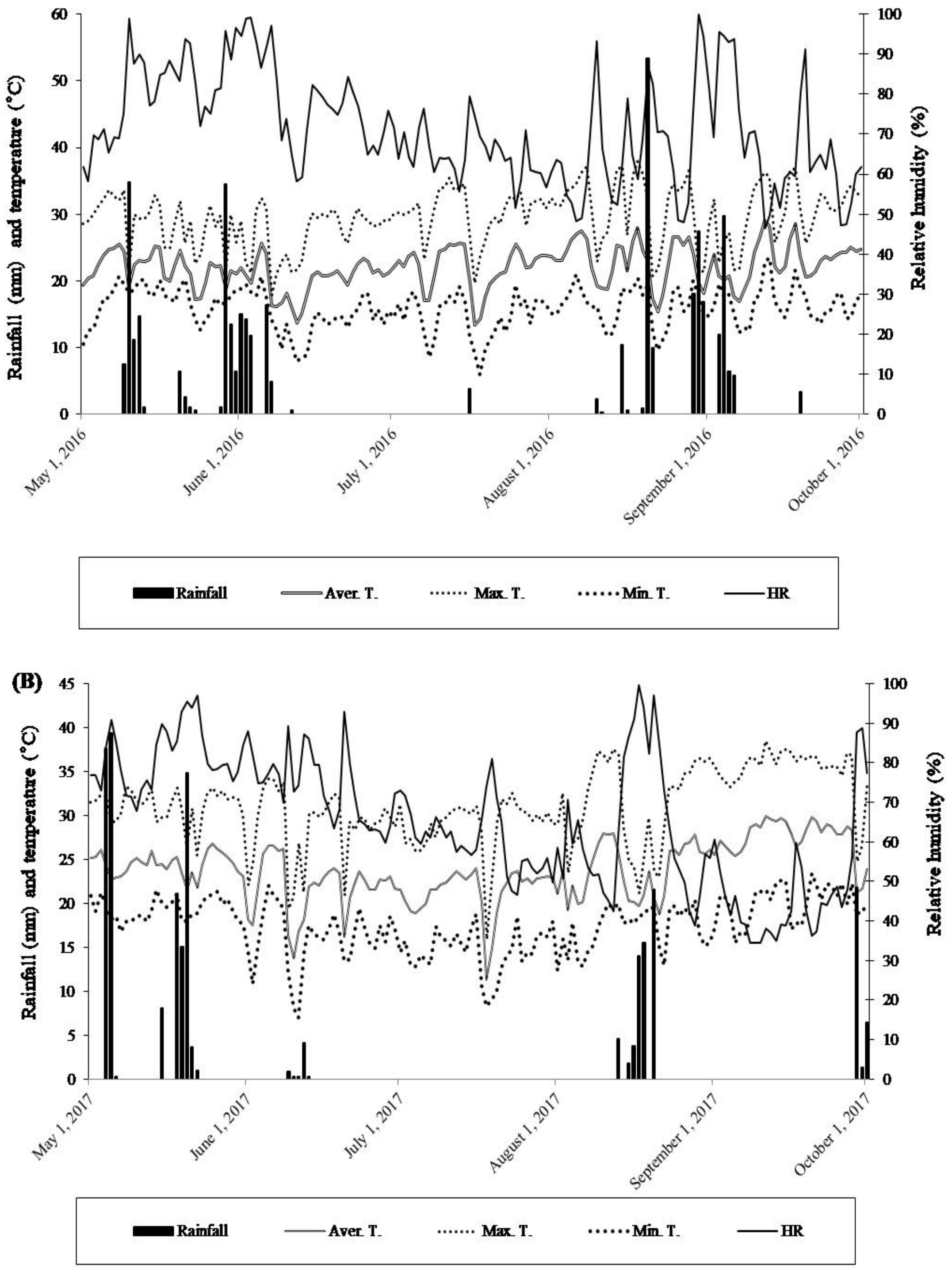

Supplementary Figure S1. Climate data during wheat cultivation in 2016 (A) e 2017 (B) at meteorological station of UNESP.
